# Percutaneous Bone Implant Surgery: A MIPS Modified Technique

**DOI:** 10.1002/lary.32192

**Published:** 2025-05-27

**Authors:** Sofia Pizzolante, Edoardo Covelli, Chiara Filippi, Maurizio Barbara

**Affiliations:** ^1^ Department of Neuroscience Mental Health and Sensory Organs (NESMOS), “Sapienza” University of Rome, “Sant'andrea” Hospital Rome Italy

**Keywords:** bone, deafness, implants, percutaneous BCI

## Abstract

Since their introduction, passive percutaneous hearing aids have undergone substantial evolution, including changes in implant production, improvements in the sound processor, and simplification of surgical implantation techniques. The latest innovation comes from the minimally invasive technique proposed for the PONTO system (MIPS), which does not involve the creation of a mucoperiosteal flap in order to leave the surrounding soft tissue and vascular microcirculation intact. This study proposes a modified surgical technique compared to the one proposed for the PONTO system in order to overcome some steps of the traditional surgical technique for the placement of the Baha Connect prosthesis. Our technique does not involve any incision but the exposure of the periosteum using a skin punch and subsequent drilling without the use of any protective cannula. The described procedure allows one to overcome some steps of the traditional surgical technique and, consequently, also some post‐operative complications. Moreover, a minimally invasive procedure can help reduce surgical time and the invasiveness of the application.

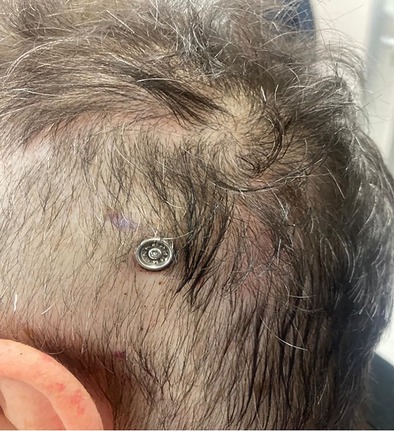

## 1 Introduction

In the last years, implantable bone conduction implants (BCI) have become a viable alternative for the rehabilitation of conductive and mixed hearing loss and in the case of unilateral sensorineural hearing loss (single‐sided deafness). This choice has been particularly adopted in those patients who do not benefit or are contraindicated for a conventional hearing aid, for example patients undergoing subtotal petrosectomy with external auditory canal closure, malformations of the external auditory canal, or failure after tympanoplasty/ossiculoplasty procedures [1].

BCI can be distinguished into percutaneous and transcutaneous devices; the latter are further divided into active and passive depending on the sound transmission mechanism used.

### 1.1 Active Transcutaneous BCI

Transcutaneous prostheses are characterized by a processor, positioned externally to the skin, that transmits sound to a magnet in contact with the bone through the intact skin.

In the active transcutaneous BCI, the transducer is directly in contact with the bone, under the intact skin, and transfers sound using an electromagnetic or piezoelectric system.

The BonebridgeTM system (MED‐EL; Innsbruck, Austria) uses an electromagnetic system and is indicated in patients with conductive or mixed hearing loss with a bone threshold better than or equal to 45 dB, or in single‐sided deafness.

The Osia 2 system (Cochlear Bone‐Anchored Solutions AB, Mölnlycke; Sweden) uses a piezoelectric system and is indicated in patients with a conductive or mixed hearing loss with a bone threshold better than or equal to 55 dB or in single‐sided deafness [2].

### 1.2 Passive Transcutaneous BCI

In passive transcutaneous device, stimulus is generated by the external processor.

The Baha Attract system (Cochlear Bone‐Anchored Solutions AB, Mölnlycke; Sweden) and the Alpha 2 MPO system (SOPHONOTM) (Medtronic; Dublin, Ireland) represent the passive transcutaneous implants available on the market.

### 1.3 Percutaneous BCI

Percutaneous implants require the use of a titanium screw that permanently penetrates the skin and bone. The screw is connected to an external processor. A process of osseointegration of the screw into the bone is required to ensure proper function of the prosthesis.

The percutaneous bone conduction prostheses currently available on the market are the PONTO system (Oticon Medical AB, Askim; Sweden) and the CochlearTM Baha Connect system (Cochlear Bone‐Anchored Solutions AB, Mölnlycke; Sweden).

Since their introduction in 1977, BCI have undergone substantial evolution, including changes in implant production, improvements in the sound processor, and the simplification of implantation techniques [3].

Percutaneous prostheses were the first to be developed, but they were beleaguered by soft tissue complications, according to Holgers classification [4]. In fact, soon after their introduction, it was hypothesized that adverse soft tissue reactions occurred because of skin movement adjacent to the point of penetration of the titanium screw. This led to the development of new surgical techniques with the reduction of soft tissue manipulation to minimize skin movement, culminating in the most recommended technique to date for the CochlearTM Baha Connect system (Cochlear Bone‐Anchored Solutions AB, Mölnlycke; Sweden), that is a linear incision, 10 mm anterior to the position of the prosthesis, which minimizes soft tissue involvement and further improves results. However, even this technique involves the creation of a mucoperiosteal flap, which is associated with a certain degree of tissue damage. For this reason, Oticon Medical AB (Askim; Sweden) has proposed a minimally invasive technique for the PONTO system, the so‐called MIPS (Minimally Invasive Ponto Surgery), which involves making a skin incision through a 5 mm circular biopsy punch in order to leave the surrounding soft tissue and vascular microcirculation intact. The MIPS technique also involves the use of a cannula, that is a stop sleeve that acts as a protector of the soft tissue during the milling process.

The aim of this study is to propose a surgical technique that consists of a modification of the way the periosteum is approached during the placement of the Baha Connect implant, using an incision with a biopsy punch as for the PONTO but without the use of a cannula in order to have a wider surgical field.

## 2 Methods

Our team has been performing bone anchored prosthesis surgery since 2000. In May 2024, this technique was used on a patient with single sided deafness in the left ear and right normoacusis. Pre‐operative audiometric tests were performed with a free‐field baha simulator device with good audiological response in the left ear. This study was reviewed and approved by Sapienza University Ethical Committee. The patient provided his written informed consent to participate in this study.

**FIGURE 1:**
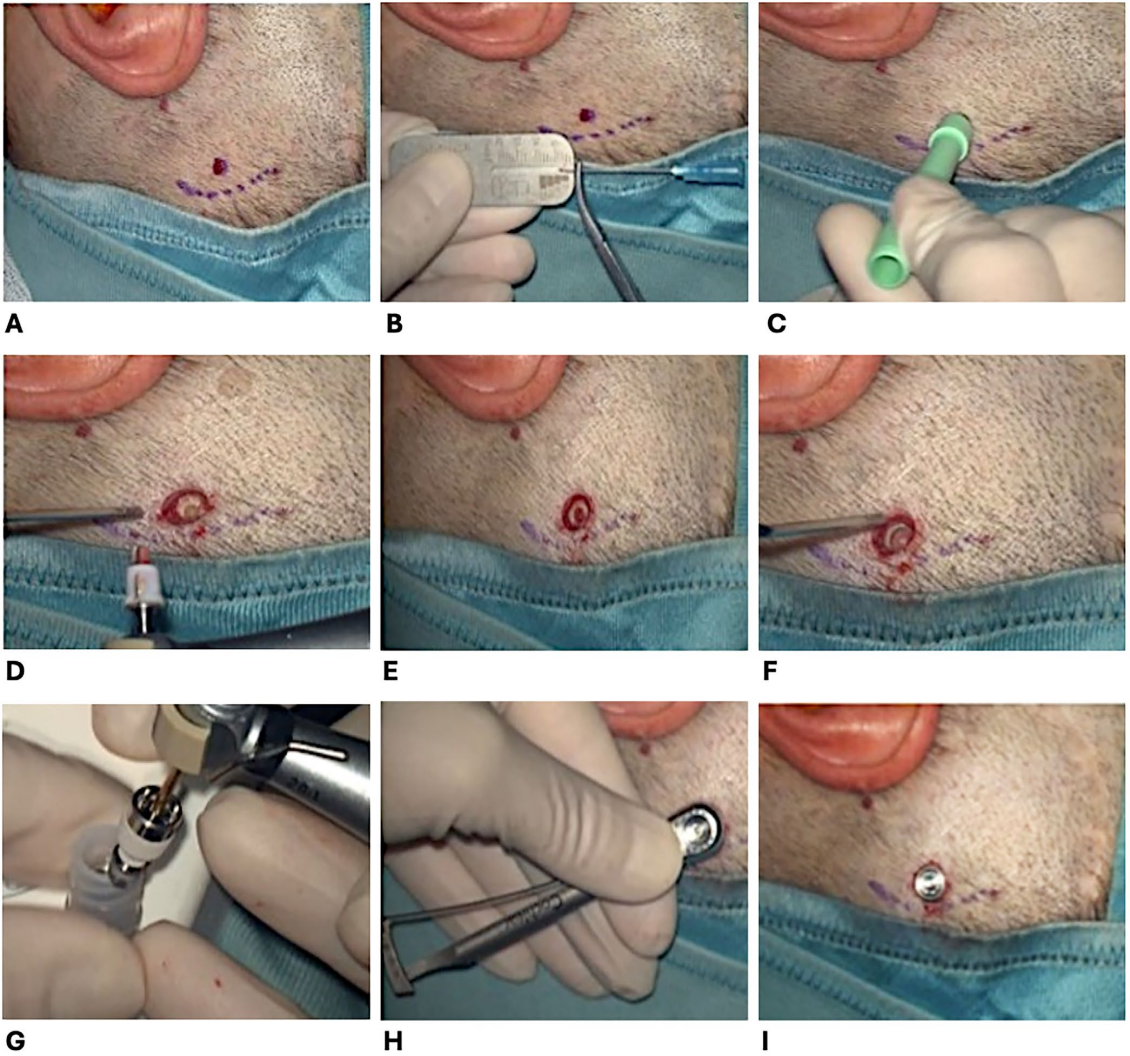
Steps for the minimally invasive percutaneous surgery. (A) The implant placement point is identified 55 mm postero‐superior to the external auditory canal. (B) The thickness between the skin and the periosteum is measured using a Baha ruler. (C) A circular incision is made with a 5 mm biopsy punch. (D) A hole is created with a 3 mm conical burr. (E) The thickness of the hole is increased with a 4 mm conical burr. (F) With an enlarging bur a countersink is made to enlarge the hole diameter. (G) The screw is positioned in the previously created hole. (H) Check that the abutment is screwed in correctly with a torque spanner. (I) Place gauze and protective cap around the prosthesis as a dressing.

The surgical technique we want to propose consists of 9 steps (Figure 1).

STEP 1: The implant placement point is identified 55 mm postero‐superior to the external auditory canal.

STEP 2: The thickness between the skin and the periosteum is measured (usually about 4–5 mm) using a hypodermic needle, forceps, and a Baha ruler.

STEP 3: After infiltrating the subcutis with an anesthetic, a circular incision is made with a 5 mm biopsy punch.

STEP 4: After removing the skin and underlying soft tissue, the periosteum is exposed, and a periosteum elevator is used to set the bone free from soft tissues, and a hole is created with a 3 mm conical bur. This technique does not involve the use of the cannula as a soft tissue protector, as in the original MIPS procedure.

STEP 5: The depth of the hole is increased with a 4 mm conical bur.

STEP 6: With an enlarging bur, a countersink is made to enlarge the hole diameter.

STEP 7: The screw is positioned in the previously created hole and screwed in with a self‐locking cutter.

STEP 8: Check that the abutment is screwed in correctly with a torque spanner.

STEP 9: Place gauze and protective cap around the prosthesis as a dressing.

## 3 Results

**FIGURE 2:**
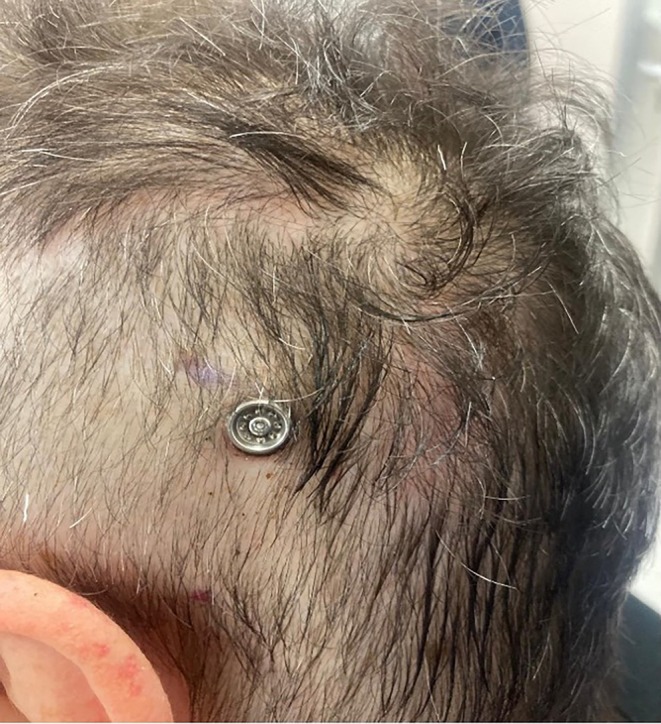
Post‐operative check‐up 1 month later.

Although the method has only been applied in one case yet, the patient is satisfied with the result obtained both from an audiological as well as an aesthetic and functional point of view. At the first post‐operative check‐up 1 month later, the prosthesis was activated. The screw was in place and the surrounding skin was undamaged (Figure 2).

## 4 Video

The minimally invasive percutaneous surgery procedure is shown in Video 1.

**VIDEO 1:** The minimally invasive percutaneous surgery procedure. Video content can be viewed at https://onlinelibrary.wiley.com/doi/10.1002/lary.32192

## 5 Discussion

The use of a minimally invasive surgical technique can help reduce surgical time and the invasiveness of the application. Compared to the technique currently proposed by the Cochlear company, which involves making a linear incision, our technique does not involve any incision but the exposure of the periosteum by means of a skin punch and subsequent milling without the use of any protective cannula. Furthermore, the absence of the cannula allows for a wider surgical field and facilitates the approach to the periosteum.

The described procedure allows overcoming some steps of the traditional surgical technique and, consequently, also some post‐operative complications: The absence of an extended surgical incision, in fact, allows having no stitches and, therefore, reduces the possibility of keloid formation. Moreover, the soft tissue removed is reduced to a minimum because it only concerns the area where the prosthesis is to be placed: this makes it possible to maintain a better integrity of the microcirculation and, therefore, reduce the healing period.

The surgical procedure described is quick and easy to reproduce. Furthermore, as stated in the Consensus Statement [5], it is a surgical procedure that can be performed under local anesthesia in cooperating adult patients and in an outpatient setting: This allows for a reduction in costs and time associated with the application of prostheses in the operating room.

## Conflicts of Interest

The authors declare no conflicts of interest.

## References

[1] M. Tisch , “Implantable Hearing Devices,” GMS Current Topics in Otorhinolaryngology, Head and Neck Surgery 16 (2017): Doc06, 10.3205/cto000145. 29279724 PMC5738935

[2] S. E. Ellsperman , E. M. Nairn , and E. Z. Stucken , “Review of Bone Conduction Hearing Devices,” Audiology Research 11, no. 2 (2021): 207–219, 10.3390/audiolres11020019. 34069846 PMC8161441

[3] S. N. Ghossaini and P. C. Roehm , “Osseointegrated Auditory Devices: Bone‐Anchored Hearing Aid and PONTO,” Otolaryngologic Clinics of North America 52, no. 2 (2019): 243–251, 10.1016/j.otc.2018.11.005. 30617010

[4] E. Verheij , A. Bezdjian , W. Grolman , and H. G. Thomeer , “A Systematic Review on Complications of Tissue Preservation Surgical Techniques in Percutaneous Bone Conduction Hearing Devices,” Otolaryngology and Head and Neck Surgery 37, no. 7 (2016): 829–837, 10.1097/MAO.0000000000001091. 27273402

[5] L. Bruschini , P. Canzi , A. Canale , et al., “Implantable Hearing Devices in Clinical Practice. Systematic Review and Consensus Statements,” Acta Otorhinolaryngologica Italica 44, no. 1 (2024): 52–67, 10.14639/0392-100X-N2651. 38165206 PMC10914359

